# Hepatitis E Virus Seroprevalence Indicated a Significantly Increased Risk Selectively in Patients with Gastric Cancer among 17 Common Malignancies

**DOI:** 10.3390/jcm12020437

**Published:** 2023-01-05

**Authors:** Xiaona Lin, Ming Luo, Qiuxiong Lin, Juan Zhang, Teng Li, Xiaoyong Pu, Keping Xie, Jun Hou, Ren Chen

**Affiliations:** 1 The Laboratory of Computational Medicine and Systems Biology, School of Medicine, South China University of Technology, Guangzhou 510006, China; 2 Guangdong Institute of Gerontology, Guangdong Provincial People’s Hospital, Guangdong Academy of Medical Sciences, Guangzhou 510080, China; 3 Department of Infectious Disease, Guangdong Provincial People’s Hospital, Guangdong Academy of Medical Sciences, Guangzhou 510080, China; 4 Laboratory Department, Guangdong Provincial People’s Hospital, Guangdong Academy of Medical Sciences, Guangzhou 510080, China; 5 Department of Urology, Guangdong Provincial People’s Hospital, Guangdong Academy of Medical Science, Guangzhou 510080, China; 6 School of Medicine, South China University of Technology, Guangzhou 510006, China

**Keywords:** hepatitis E virus seroprevalence, cancers, risk, common malignant tumors

## Abstract

Background: The impact of hepatitis E virus (HEV) infection on cancer development has been poorly investigated. This study aimed to explore the relationship between HEV seroprevalence and cancer risks and to identify high cancer risk subgroups in HEV-exposed populations. Methods: HEV seroprevalence status was determined in cancer and non-cancer subjects. Logistic regression and sensitivity analyses were used to assess the relationship between HEV antibody seropositivity and cancer risk for 17 cancer types. Additionally, interaction analyses were applied to interpret the association of HEV seroprevalence and other cancer risk factors. Results: Of the enrolled 4948 cancer and 4948 non-cancer subjects, cancer subjects had a higher anti-HEV seropositivity than non-cancer subjects (46.36% vs. 32.50%, *p* < 0.01). However, this divergency varied in degrees across different cancer types. Additionally, HEV seroprevalence was associated with cancer risk in young males (OR: 1.64, 95% CI: 1.19–2.27, *p* < 0.01). Remarkably, a significant association between HEV seroprevalence and cancer risk was observed only in gastric cancer patients (OR: 1.82, 95% CI: 1.07–3.09, *p* = 0.03). Conclusions: HEV seroprevalence was associated with cancer risk selectively in gastric cancer patients and young males, suggesting that cancer screening, particularly gastric cancer, should be regularly performed in young males with a history of HEV exposure.

## 1. Introduction

The annual hepatitis E virus (HEV) infection rate is approximately 20 million globally [1]. The prevalence of HEV infection varies largely across geographical and occupational settings. Previous studies revealed that the HEV antibody seropositivity rate in the general population ranges from 2% to 77.7% in Southeastern Asia and from 2.17% to 52.2% in European countries [2,3,4]. In China, the estimated prevalence of HEV infection in the general population is from 13% to 14.22% in some provinces [5,6]. However, a much higher anti-HEV seropositivity rate (38.34%) was reported in the population in Guangdong, China [7]. Our previous study also demonstrated a relatively high HEV seropositivity rate in both hepatocellular carcinoma (HCC) patients (40.93%) and healthy individuals (41.81%) from Guangdong [8]. However, there are no comprehensive estimations of HEV seroprevalence in cancer patients or in each common cancer type.

Cancer is a leading cause of death worldwide, and 12–15% of cancer cases are attributable to chronic viral infection [9,10]. HEV is both an enveloped and non-enveloped single-stranded RNA virus with a genome size of 7.2 kb [11]. Up to now, four main HEV genotypes have been identified. HEV genotypes 1 and 2 are transmitted through a fecal–oral route and only infect humans, while HEV genotypes 3 and 4 are able to be spread from animals to human beings [12]. The main site of HEV replication is the liver as well as extrahepatic organs, including the small intestine, colon, spleen, stomach, and kidney [11]. Acute or chronic infection can directly damage liver cells, resulting in impaired liver function. Previous studies indicated that HEV replicated in the hepatocyte cytoplasm and, afterwards, was released into the bile and blood [13]. Moreover, HEV infection may cause liver damage due to immune regulation from natural killer (NK) cells and cytotoxic T cells [14]. On the other hand, extrahepatic infection can cause neurological, renal, gastrointestinal, and hematological disorders [15]. Nevertheless, the detailed pathogenesis of hepatitis E remains largely unknown. A previous study revealed that around 26% of cancer patients in eastern China were infected with HEV and that the prevalence of HEV in them was much higher than that in non-cancer patients, suggesting a positive correlation between HEV infection and cancer development, including extrahepatic cancers [6]. Our previous study also revealed that HEV exposure might abate the detrimental effect of HBV on HCC, indicating that the association between HEV exposure and cancer might be modified by other factors [8]. Nonetheless, there exists only very limited epidemiological information on the interaction between HEV exposure and other cancer risk factors in cancer populations, including HCC and extrahepatic cancers.

Collectively, we hypothesized that HEV infection was associated with cancer risk in some extrahepatic organs and that the association between HEV and cancer risk can be modified by other significant oncogenic factors. Therefore, we conducted a current, single-center, case-control study to address these important questions.

## 2. Materials and Methods

### 2.1. Study Design

**Figure 1:**
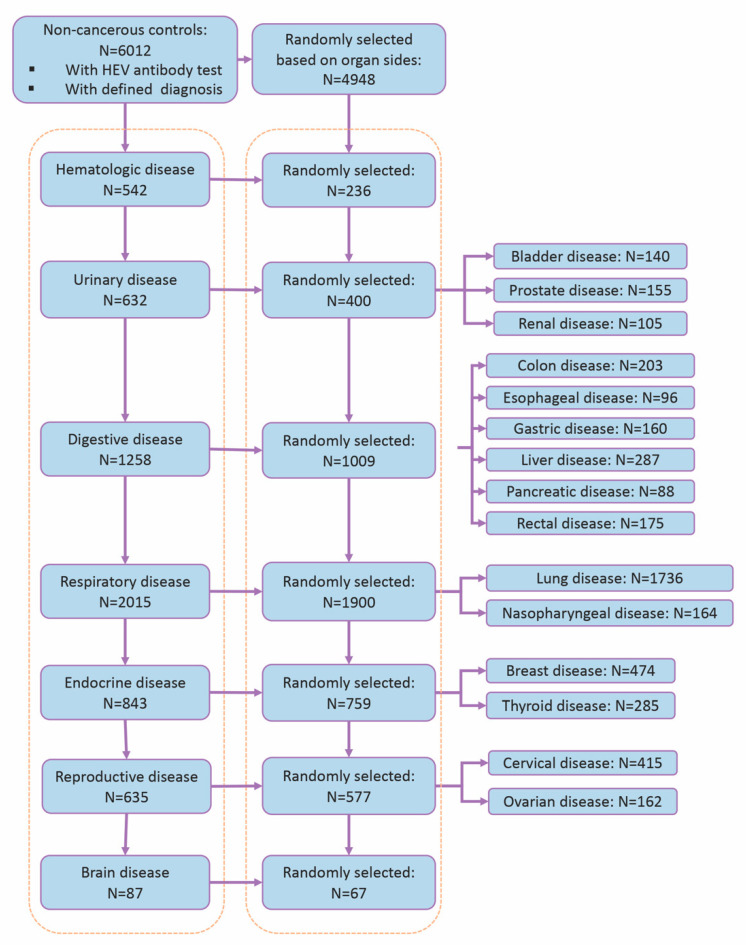
Flowchart of selection of controls in the study.

We performed a retrospective case-control study. A review board approval and a waiver of informed consent were obtained from Guangdong General Hospital (KY-Q-2021-131-02). All cancer inpatients who were admitted into the selected departments in Guangdong General Hospital between 1 January 2019 and 31 October 2020 and had an HEV virological test were enrolled in the study. In total, patients diagnosed with the 17 top common cancer types based on global cancer statistics information were enrolled, including both liver cancer and extrahepatic cancers. Extrahepatic cancers included hematologic malignancies, bladder cancer, prostate cancer, renal cancer, colon cancer, esophageal cancer, gastric cancer, pancreatic cancer, rectal cancer, lung cancer, nasopharyngeal cancer, breast cancer, thyroid cancer, cervical cancer, ovarian cancer, and brain cancer. The diagnoses were determined based on clinical symptoms, computerized tomography (CT), magnetic resonance imaging (MRI), ultrasound findings, and pathological results and recorded according to the International Classification of Diseases, 10th Revision (ICD-10). Cancer patients who were admitted multiple times during that period were treated as single entries, and the primary diagnoses of the patients were recorded in the study. Following this strategy, 4948 eligible subjects with cancers that originated from 17 different organs were included in this study. The control subjects were patients who had to meet the following criteria. First, in order to reduce the bias of hospital-acquired infection between departments, the controls were admitted for non-cancerous diseases located at the same 17 organs, respectively. Second, they were tested for anti-HEV antibodies at admission. Last, they were admitted during the same period as the cancer subjects. Patients with the following diseases were excluded from the control group: any type of cancer, other viral infection, autoimmune diseases, end-stage disease, and tuberculosis. As a result, 6012 non-cancerous controls were eligible for the inclusion criteria. Out of these control subjects, random selections were performed to make sure that each cancer subgroup, stratified by cancer sites, had a similar number of corresponding control cases (Figure 1). Finally, 4948 controls were included for further analyses.

### 2.2. Data Collection

Demographic and anthropometric information of the included subjects was retrieved from clinical records. Medical comorbidities, such as hypertension, diabetes, coronary heart disease, and hepatitis viral infection, were collected at baseline. The recorded personal life behavior and occupational information were also obtained. All of the above information was applied as covariates in the analyses.

### 2.3. Detection of HEV Seroprevalence

By far, HEV infection has not been well defined [16]. HEV seroprevalence was commonly used as the evidence of HEV exposure and infection in epidemiological studies [17,18,19]. Because the aim of this study was to evaluate the impact of HEV exposure, including recent exposure and repeated exposure, on cancer risk, we defined the presence of anti-HEV Ig M and/or IgG as anti-HEV seropositivity [8]. Both anti-HEV IgM and IgG were detected by an automated system (F.A.M.E. microplate processor) using a Wantai testing kit (Wantai, Beijing, China).

### 2.4. Statistical Analyses

In this study, a Shapiro–Wilk test was applied to test the distribution of the variables. Continuous variables were summarized as mean or median based on the distribution. A t-test and Wilcoxon test were used to measure differences between the two groups in normal distribution and non-normal distribution variables, respectively. Meanwhile, we measured the categorical variables by percentage (%), and the associations were tested by a chi-squared or Fisher’s exact test depending on the sample sizes. We compared the seroprevalence of anti-HEV antibodies in cancer patients and control subjects. Crude and adjusted odd ratios (ORs) were estimated by a logistic regression model. Adjusted ORs were generated by adjusting age, gender, weight, smoking, alcohol drinking, family cancer history, hypertension, diabetes, coronary heart disease history, occupation, and HBV infection history. Sensitivity analyses were performed to show the robustness of the results by excluding smokers, alcohol addicts, and patients with hypertension, diabetes, coronary heart disease, family cancer history, and HBV infection. Additionally, logistic regression was used to evaluate the multiplicative interaction. Additive interaction between any two potential risk factors was assessed through the bootstrap method by calculating relative excess risk ratio (RERI), attributable ratio (AP), interaction index (S), and 95% confidence interval (CI) [20,21]. No additive interaction was considered if the 95% CI of RERI and S included 1 and the 95% CI of AP included 0. “Interaction R” R package was used in multiple and additive interaction analyses.

As only 33.62% of the patients had “height” recorded, this parameter was excluded from the analysis. The proportions of the missing values of the other 13 variables were between 0 and 7.17%. “Multivariate Imputation by Chained Equations” (MICE) R package was applied to impute the missing data [22]. Five multiple imputed datasets were created, and the estimates based on each imputed dataset were combined according to Rubin’s rules. We used *p* < 0.05 (2-tailed) to define statistical significance and R program (version 4.1.0) for all analyses.

## 3. Results

### 3.1. HEV Seropositivity Rates Were Higher in the Cancer Group Than Those in the Corresponding Non-Cancer Group in Guangdong

**Figure 2:**
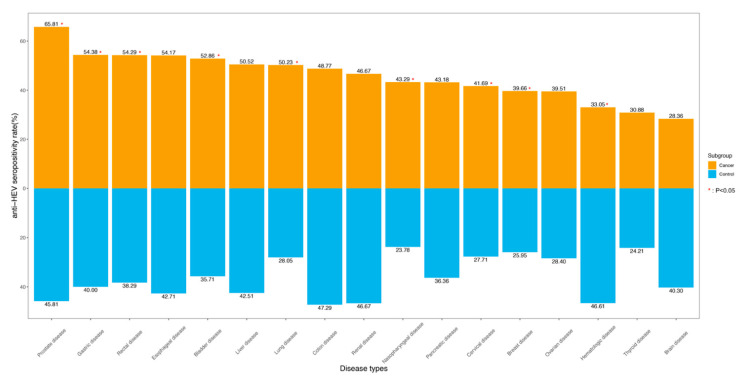
Anti-HEV seropositivity rates among cancer patients and non-cancer patients based on 17 organ sites.

This study enrolled 4948 cancer cases and 4948 non-cancer patients as controls. The rate of HEV seropositivity was higher in the cancer groups than that in the control groups (46.36% vs. 32.50%, *p* < 0.01). When the cancers were divided by organ sites, a significant difference in the positivity of anti-HEV antibodies was not detected between HCC and non-HCC liver diseases. However, a significantly high prevalence of anti-HEV antibodies was detected in a few types of extrahepatic cancers as compared to the corresponding control groups (Figure 2), including the majority of the studied solid tumors, such as cancers in the urinary tract, digestive tract, and respiratory system. Interestingly, HEV seroprevalence in patients with hematologic malignancies was lower than that in the control subjects (33.05% vs. 46.61%, *p* < 0.01).

Furthermore, among all studied extrahepatic malignancies, HEV seroprevalences varied among cancer types. For example, patients with urinary cancer showed the highest HEV seroprevalence (56.25%) followed by cancers in the digestive system (51.14%) and respiratory system (49.63%). On the other hand, patients with brain cancer (28.36%) were the least likely to be infected with HEV, while patients with hematological malignancies (33.05%) and those with cancers exhibiting endocrine features (36.36%) had moderate levels of HEV seropositivity. Collectively, more than half of the patients with the following cancers had a positive test for HEV antibodies: prostate cancer (65.81%), gastric cancer (54.38%), rectal cancer (54.29%), esophageal cancer (54.17%), bladder cancer (52.86%), and lung cancer (50.23%) in addition to liver cancer (50.52%).

Remarkably, our results indicated that the rate of HEV seropositivity was also high in patients with the following non-cancerous diseases: hematologic (46.61%), urologic (42.50%), digestive (41.82%), and cerebral disorders (40.30%).

### 3.2. HEV Seroprevalence Was Associated with an Increased Risk in Gastric Cancer

**Figure 3:**
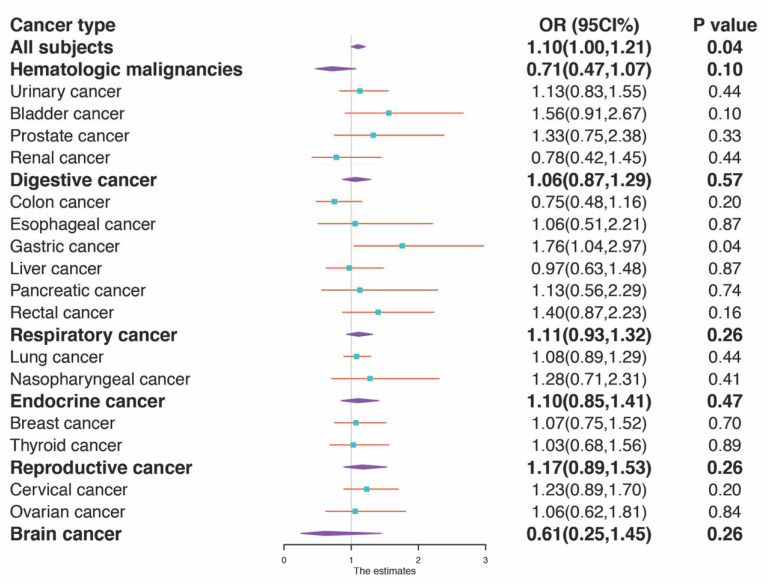
A forest plot of the association between HEV anti-seropositivity and the risk of cancer. A forest plot for HEV seropositivity and the risk of cancer is presented by cancer subtypes. Subtype-specific odds ratios (OR) adjusted by all studied risk factors and 95% confidence interval (95% CI) are denoted by cyan boxes. The *p* value for OR by subtype is shown as well.

As shown in Table 1 and Figure 3, when analyzing all cancer cases together, HEV seropositivity was associated with a slightly elevated risk of cancer development even after adjusting for age, gender, weight, smoking, alcohol drinking, family cancer history, hypertension, diabetes, coronary heart disease history, occupation, and HBV infection history (OR: 1.10, 95% CI: 1.00–1.21, *p* = 0.04). Notably, this association remained significant only in gastric cancer, when cancers were divided into different groups by organ sites after risk factors were adjusted (OR: 1.82, 95% CI: 1.07–3.09, *p* = 0.03). More importantly, the association between HEV seropositivity and gastric cancer risk was verified robustly by a sensitivity analysis, in which the participants with hypertension, diabetes, smoking, drinking, HBV infection, family cancer history, and coronary heart disease were successively excluded (Table 2). However, no significant association between HEV seroprevalence and cancer risks was observed in other extrahepatic cancers. Again, HEV infection was not identified as a risk factor for liver cancer (Table 1).

### 3.3. The Association between HEV Seroprevalence and Cancer Risk Can Be Modified by Age and Gender

In order to explore the potential interaction between HEV exposure and other cancer risk factors, we tested the multiplicative interaction and addictive interaction between HEV and widely accepted oncogenic factors (Table 3 and Table 4). The impacts of both multiplicative and additive interactions on cancer risk were observed between HEV prevalence and age (OR: 0.98, 95% CI: 0.98–0.99, *p* < 0.01; RERI: 0.04, 95% CI: 0.02–0.06; AP: 0.01, 95% CI: 0.01–0.02; S: 1.02; 95% CI: 1.02–1.03) and anti-HEV antibody seropositivity and gender (OR: 1.19, 95% CI: 1.01–1.41, *p* = 0.03; RERI: 0.35, 95% CI: 0.10–0.60; AP: 0.17, 95% CI: 0.06–0.29; S: 1.53; 95% CI: 1.09–2.15). Nonetheless, there were no statistically significant interactions between HEV seropositivity and HBV infection, smoking, and alcohol drinking or their impacts on promoting cancer development.

### 3.4. HEV Seroprevalence Was Independently Associated with Cancer Risk in Young Males

After further adjustment for potential confounders, an elevated cancer risk in male patients who were infected with HEV and younger than 45 years old was detected (OR: 1.64, 95% CI: 1.19–2.27, *p* < 0.01) (Table 5). Moreover, HEV seropositivity as a cancer risk factor in young males was further tested and confirmed by sensitivity analyses, in which patients with hypertension, diabetes, smoking, drinking, HBV infection, family cancer history, and coronary heart diseases were successively excluded. However, HEV seropositivity was not a cancer risk in female and elderly male cancer patients (Table 6).

## 4. Discussion

In this study, we found a robust association between HEV seroprevalence and gastric cancer risk. We also found that HEV seroprevalence was associated with an elevated risk of cancer development in male patients younger than 45 years old. However, no modification effect between HEV and HBV, HEV and smoking, or HEV and alcohol on cancer development was discovered in our study.

HEV exposure was more commonly observed in the cancer patients as compared to non-cancer patients [6,23]. There are a few possible explanations. First, cancer patients are immunocompromised and unable to form a potent immune response against HEV. Second, similar to other viruses, HEV may drive or accelerate carcinogenesis by causing genome instability mediated by persistent inflammation [24]. Third, aging is regarded as one of the most important cancer risk factors. In our study, we observed that cancer patients were significantly older than non-cancer patients (55.61 vs. 43.15 years) (Table S1). A U.S. study revealed that increased age was the only factor associated with HEV seropositivity, probably a consequence that resulted from repeated HEV exposure and accumulated antibodies over time [18]. Collectively, these mechanisms might lead to more HEV exposure prevalence in cancer patients.

In our study, HEV seroprevalence was correlated with a slightly elevated cancer risk in all cancer patients. However, this association was not statistically significant as determined by the sensitivity analyses (Table S2). Mara et al. demonstrated that HEV was involved in lots of vital cancer pathways related to apoptosis, oxidative stress, proliferation, growth and angiogenesis [25]. By disrupting the cell signal of tumor suppressor proteins or upregulating the proliferation pathways, HEV promotes cancer development. Moreover, chronic inflammation is a fundamental factor for promoting the oncogenesis process. HEV invasion led to continuous cell death and inflammatory cell infiltration, after which the active immune response was initiated and a series of oncogenic processes were stimulated. Our observation might result from the heterogeneity in cancer types, that is, in other words, HEV plays differential roles in oncogenesis in different cancers. We performed a subgroup analysis to further test whether HEV seropositivity was a risk factor in individual cancer types. Previous research revealed that tissue-specific factors secreted by stromal cells would influence the outcome of immune response to different pathogens in different tissues [26]. Moreover, age associates differentially with different cancers, which, in turn, are linked to diverse degrees of HEV seropositivity. Therefore, it is highly possible to observe inconsistent degrees of HEV prevalence among different cancer types.

Moreover, the interactions of HEV exposure and other common oncogenic risk factors in cancer were explored. We discovered that age and gender, but not HBV infection, smoking, and alcohol intake, would be the modification factors on the association between HEV and cancer risk. We then stratified patients by age and gender to test their specific effects. Remarkably, we found that HEV seroprevalence was indicated as a significant cancer risk factor in young male patients but not in elderly male patients and all female patients. We speculated that the change of hormonal levels due to aging may contribute to some extent to this gender- and age-related discrepancy. Higher progesterone and estradiol levels were indicated to predispose individuals to HEV exposure according to previous studies [27]. In addition, estrogens largely enhance immune functions while androgens mainly suppress immune effects [28]. Thus, young female patients were protected by the hormone when they were infected with HEV. Therefore, we observed an increased risk between HEV seropositivity and cancer incidence in young male but not in young female groups. The endocrine and immune systems change with advancing age, especially among females. A protective effect of estrogen in menopausal women was reduced; a similar trend but not a significant risk effect was observed between HEV exposure and cancer risk among male and female old patients.

Aging imposes a complicated impact on the immune response against virus infection with respect to gender. It is well documented that somatic mutations increase with aging, their patterns vary between the two genders, and the effect on the immune system exerted by aging is divergent between sexes. Meanwhile, growing evidence has suggested that the tumor immune microenvironment has a remarkable impact on the differences in disease development and prognosis between males and females [29]. These studies directly and indirectly provide theoretic support for the summative effects on the different associations between HEV and cancer risks among different gender and age subgroups. Nevertheless, the potential mechanisms are needed to be further addressed.

Interestingly, HEV infection was not linked to HCC development based on the current study. The liver is the vital organ for HEV residence and replication. Acute and chronic HEV infection directly damages liver cells; therefore, HEV infection has been suspected to be a risk factor of liver cancer. The postulation is supported neither by the current study nor our previous study [8]. More interestingly, the group analyses found that HEV was statistically significantly associated with an elevated cancer risk in one type of extrahepatic malignancy, gastric cancer. To further evaluate the probability of HEV as a cancer risk factor, we adjusted the analyses by excluding the patients with different diseases successively in sensitivity analyses to eliminate the confounding effects induced by the co-existing comorbidities. Additionally, the analyses revealed that the association between HEV seroprevalence and gastric cancer risk was robust and not modified by other comorbidities.

Although the detection of HEV in the stomach has been reported [11], the mechanisms of gastric damage caused by HEV infection were largely unknown. Previous animal studies revealed that HEV RNA was present in the small intestine and stomach in rabbits, implying that the fecal–oral transmission of HEV might replicate with a high possibility in the gastro-intestinal tract and trigger a wide range of oncogenesis processes [30]. In addition, previous investigations indicated that certain host factors such as HSPG, GRP78, and ATP5B were involved in the cell entry of HEV [31]. Moreover, GRP78 and ATP5B were expressed in normal gastric tissue and were good prognostic factors of gastric cancer according to the HPA (Human Protein Atlas) database and previous reports [32,33]. Thus, we have rationale to speculate that HEV infects gastric tissue by binding to receptors such as GRP78 and ATP5B and contributes to gastric cancer development. Nevertheless, further studies are warranted to explore the mechanisms underlying the increased risk of gastric cancer development in individuals infected with HEV.

Finally, we found a relatively weak association between HEV seropositivity and hematological malignancies in comparison with other cancers. Only limited studies have demonstrated that HEV may elevate the mortality and liver-related morbidity in patients with hematological malignancies [34]. As blood-borne transmission is recognized as a main HEV exposure source in hematological malignancy, we collected the transfusion information of hematological patients. Our results showed that there was no significant difference in the transfusion frequencies between malignant cases and non-malignant cases. In our study, a relatively high positivity rate of anti-HEV antibodies was also observed in the cases with non-malignant hematological disorders. Even though a high HEV seropositivity was reported in blood donors, HEV was not routinely detected currently in China before blood transfusion [5]. Therefore, blood recipients, especially when they have hematological diseases, are at a high risk of HEV exposure through the route of blood transfusion.

However, our study has several limitations. First, this is a single-center study. The patients included might not be representative of all cancer and non-cancer patients in China since they might experience unique environmental influences and lifestyles. Therefore, future multi-center studies should be performed to validate our findings. Second, for some specific cancers, certain risk factors were not well documented, such as Helicobacter pylori in gastric cancer. Unfortunately, a Helicobacter pylori test was not regularly conducted in hospitalized patients in China due to the health economic concern, and we cannot include the results of Helicobacter pylori in this study. Wang et al. illustrated that the infection of Helicobacter pylori was related to chronic hepatitis B [35]. However, the association between Helicobacter pylori and HEV was not addressed and should be addressed in the future. Third, the HEV seropositivity rates of non-cancer hospital controls in this study were higher than those in other areas of China [6]. However, since HEV antibody tests were seldomly conducted in healthy individuals, we could not include enough control samples of healthy populations to further compare the seroprevalence between healthy individuals and patients with non-cancerous diseases. Epidemiology studies should be designed to address HEV-exposed distribution issues based on various geographic areas and population groups in the future. Moreover, similar to other retrospective studies, a temporal sequence of HEV exposure and cancer development could not be confirmed. Therefore, we cannot claim a conclusive cause–effect relationship between HEV exposure and cancer risk. Future prospective studies on this subject may be useful but certainly time consuming, while new epidemiology methods, such as Mendelian randomization, could be alternatives to address this important question.

## 5. Conclusions

Our study provided epidemiological evidence that there was a strong association between high HEV seroprevalence and cancer risk in males as well as between HEV seroprevalence and gastric cancer risk. Our results highlight the importance of more in-depth studies to clarify the risk factors of HEV infection and to investigate the underlying pathways through which HEV infection causes a higher risk of cancers.

## Figures and Tables

**Table 1:** Association between HEV exposure and risk of all cancers.

	Subject Number *	Crude Model		Adjusted Model 1 ^#^		Adjusted Model 2 ^$^	
		OR (95% CI)	*p* Value	OR (95% CI)	*p* Value	OR (95% CI)	*p* Value
All organ sites	9896	1.80 (1.65, 1.95)	<0.01	1.12 (1.02, 1.22)	0.02	1.10 (1.00, 1.21)	0.04
Hematopoietic system	472	0.57 (0.39, 0.82)	<0.01	0.70 (0.47, 1.05)	0.08	0.71 (0.46, 1.08)	0.11
Urinary tract	800	1.74 (1.31, 2.30)	<0.01	1.11 (0.81, 1.50)	0.52	1.14 (0.83, 1.56)	0.42
Bladder cancer	280	2.02 (1.25, 3.26)	<0.01	1.47 (0.88, 2.45)	0.14	1.55 (0.90–2.65)	0.11
Prostate cancer	310	2.28 (1.44, 3.60)	<0.01	1.23 (0.67, 2.28)	0.5	1.22 (0.64–2.32)	0.55
Renal cancer	210	1.00 (0.58, 1.72)	1	0.79 (0.44, 1.41)	0.42	0.77 (0.41, 1.45)	0.42
Digestive tract	2018	1.46 (1.22, 1.74)	<0.01	1.09 (0.91, 1.32)	0.34	1.06 (0.87, 1.29)	0.55
Colon cancer	406	1.06 (0.72, 1.57)	0.77	0.78 (0.51, 1.19)	0.25	0.75 (0.48, 1.16)	0.2
Esophageal cancer	192	1.59 (0.90, 2.80)	0.11	1.11 (0.59, 2.10)	0.75	1.07 (0.51–2.25)	0.86
Gastric cancer	320	1.79 (1.15, 2.79)	0.01	1.37 (0.86, 2.19)	0.19	1.82 (1.07–3.09)	0.03
Liver cancer	574	1.38 (0.99, 1.92)	0.06	0.96 (0.67, 1.39)	0.84	0.95 (0.62–1.45)	0.81
Pancreatic cancer	176	1.33 (0.73, 2.44)	0.36	1.12 (0.58, 2.18)	0.73	1.09 (0.53–2.25)	0.81
Rectal cancer	350	1.87 (1.48–2.35)	<0.01	1.26 (0.98–1.64)	0.07	1.19 (0.92–1.55)	0.19
Respiratory system	3800	2.57 (2.25, 2.95)	<0.01	1.13 (0.96, 1.33)	0.15	1.11 (0.93, 1.32)	0.25
Lung cancer	3472	2.59 (2.25, 2.98)	<0.01	1.11 (0.93, 1.31)	0.25	1.08 (0.90–1.30)	0.43
Nasopharyngeal cancer	328	2.45 (1.52–3.93)	<0.01	1.22 (0.70, 2.11)	0.49	1.29 (0.71–2.36)	0.4
Endocrine system	1518	1.69 (1.35, 2.10)	<0.01	1.16 (0.91, 1.48)	0.22	1.10 (0.86, 1.42)	0.45
Breast cancer	948	1.88 (1.42–2.47)	<0.01	1.10 (0.78, 1.55)	0.57	1.08 (0.76–1.53)	0.68
Thyroid cancer	570	1.40 (0.97, 2.02)	0.08	1.09 (0.73, 1.62)	0.68	1.04 (0.69–1.58)	0.85
Reproductive system	1154	1.80 (1.41, 2.30)	<0.01	1.24 (0.95, 1.62)	0.12	1.17 (0.89, 1.53)	0.27
Cervical cancer	830	1.86 (1.39, 2.49)	<0.01	1.32 (0.96, 1.81)	0.09	1.22 (0.88–1.69)	0.23
Ovarian cancer	324	1.65 (1.03–2.62)	0.04	1.06 (0.63–1.77)	0.83	1.08 (0.63–1.85)	0.79
Brain	134	0.59 (0.29, 1.21)	0.15	0.62 (0.27, 1.38)	0.24	0.53 (0.21, 1.30)	0.16

* Cancer cases and controls are matched by 1:1. ^#^ Adjusted by age and gender; ^$^ adjusted by age, gender, weight, occupation, hypertension, diabetes, smoking, alcohol drinking, HBV infection history, family cancer history, and coronary heart disease history. The overall results of each organ system are denoted by grey color. Abbreviations: OR = odds ratio; 95% CI = 95% confidence interval.

**Table 2:** Sensitivity analyses on the association of HEV exposure and cancer risk in patients with gastric cancer.

	Subject Number	Crude Model		Adjusted Model #	
		OR (95% CI)	*p* Value	OR (95% CI)	*p* Value
Excluding participants with hypertension	255	2.48 (1.49–4.13)	0.03	2.54 (1.37–4.72)	<0.01
Excluding participants with diabetes	284	1.84 (1.15–2.95)	0.01	1.92 (1.1–3.35)	0.02
Excluding participants of smoker	282	1.75 (1.09–2.81)	0.02	1.83 (1.04–3.19)	0.04
Excluding participants of drinker	309	1.85 (1.18–2.92)	<0.01	1.87 (1.09–3.21)	0.02
Excluding participants with HBV infection	303	1.76 (1.1–2.84)	0.02	1.9 (1.07–3.37)	0.03
Excluding participants with family cancer history	315	1.76 (1.12–2.75)	0.01	1.78 (1.04–3.03)	0.04
Excluding participants with coronary heart disease	305	1.73 (1.08–2.8)	0.02	1.70 (1.00–3.02)	0.04

# Adjusted by age, gender, weight, occupation, hypertension, diabetes, smoking, alcohol drinking, HBV infection history, family cancer history, and coronary heart disease history. Abbreviations: OR = odds ratio; 95% CI = 95% confidence interval.

**Table 3:** Multiplicative interaction between HEV and other cancer risk factors in all cancer patients.

	OR (95% CI)	*p* Value
HEV + Age	0.98 (0.98–0.99)	<0.01
HEV + Gender (male)	1.19 (1.01–1.41)	0.03
HEV + HBV infection	0.99 (0.64–1.53)	0.97
HEV + Smoking	1.00 (0.75–1.33)	1.00
HEV + Alcohol drinking	0.96 (0.60–1.54)	0.87

**Table 4:** The additive interactions between HEV and other cancer risk factors among all cancer patients.

	RERI		AP		S	
	Point Estimate	95% CI	Point Estimate	95% CI	Point Estimate	95% CI
HEV + Age	0.04	0.02, 0.06	0.01	0.01, 0.02	1.02	1.02, 1.03
HEV + Gender	0.35	0.10, 0.60	0.17	0.06, 0.29	1.53	1.09, 2.15
HEV + HBV infection	0.94	−0.56, 2.45	0.24	−0.07, 0.54	1.46	0.84, 2.51
HEV + Smoking	1.35	0.20, 2.50	0.26	0.08, 0.45	1.49	1.08, 2.06
HEV + Alcohol drinking	0.43	−0.67, 1.52	0.15	−0.19, 0.49	1.29	0.68, 2.43

Abbreviations: OR = odds ratio; 95% CI = 95% confidence interval.

**Table 5:** Stratified analyses by age and gender on the association of all cancer risk with HEV exposure.

		Subject Number	Crude Model		Adjusted Model #	
			OR (95% CI)	*p* Value	OR (95% CI)	*p* Value
Male	All cases	5278	1.94 (1.72, 2.18)	<0.01	1.11 (0.96, 1.27)	0.15
	Age < 45 years	2093	2.10 (1.57, 2.81)	<0.01	1.64 (1.19, 2.27)	<0.01
	Aged ≥ 45 years	3185	1.04 (0.90, 1.21)	0.56	0.98 (0.84, 1.14)	0.78
Female	All cases	4618	1.62 (1.45, 1.82)	<0.01	1.09 (0.97, 1.24)	0.15
	Age < 45 years	1356	1.43 (1.12, 1.81)	<0.01	1.00 (0.77, 1.30)	1.00
	Aged ≥ 45 years	3262	1.07 (0.93, 1.24)	0.35	1.06 (0.91, 1.22)	0.46

# Adjusted by weight, occupation, hypertension, diabetes, smoking, alcohol drinking, HBV infection history, family cancer history, and coronary heart disease history. Abbreviations: OR = odds ratio; 95% CI = 95% confidence interval.

**Table 6:** Sensitivity analyses on the association of HEV exposure and all cancer risk among males younger than 45 years.

	Subject Number	Crude Model		Adjusted Model #	
		OR (95% CI)	*p* Value	OR (95% CI)	*p* Value
Excluding participants with hypertension	2046	2.28 (1.69, 3.07)	<0.01	1.75 (1.26, 2.44)	<0.01
Excluding participants with diabetes	2081	2.12 (1.58, 2.85)	<0.01	1.61 (1.16, 2.23)	<0.01
Excluding participants of smoker	2085	2.28 (1.66, 3.13)	<0.01	1.67 (1.18, 2.36)	<0.01
Excluding participants of drinker	2092	2.05 (1.52, 2.77)	<0.01	1.53 (1.10, 2.15)	0.01
Excluding participants with HBV infection	2029	2.06 (1.50, 2.82)	<0.01	1.56 (1.11, 2.20)	0.01
Excluding participants with family cancer history	2067	2.11 (1.56, 2.85)	<0.01	1.62 (1.17, 2.25)	<0.01
Excluding participants with coronary heart disease	2092	2.12 (1.56, 2.88)	<0.01	1.64 (1.17, 2.29)	<0.01

# Adjusted by age, gender, weight, occupation, hypertension, diabetes, smoking, alcohol drinking, HBV infection history, family cancer history, and coronary heart disease history.

## Data Availability

The data used to support the findings of the study are available from the corresponding author upon request.

## Supplementary Materials

The following supporting information can be downloaded at: https://www.mdpi.com/article/10.3390/jcm12020437/s1, Table S1: Clinical information in cancer and non-cancer patients; Table S2: Sensitivity analysis on the association of HEV infection and cancer risk in all patients.
